# Distinct long-term neurological trajectories and their predictors in cerebral venous sinus thrombosis: a latent class mixed model analysis

**DOI:** 10.3389/fnhum.2026.1747667

**Published:** 2026-02-19

**Authors:** Wei Ye, Qin Zhang, Xiaoyu Chen

**Affiliations:** 1 Department of Radiology, The Affiliated Huai’an Hospital of Xuzhou Medical University, Huai’an, China; 2 Department of Pediatric Orthopedics, Shanghai Xinhua Hospital Affiliated to Shanghai Jiaotong University School of Medicine, Shanghai, China

**Keywords:** cerebral venous sinus thrombosis (CVST), latent class mixed models (LCMM), long-term follow-up, neurological trajectories, outcomes

## Abstract

**Background:**

The long-term neurological recovery of patients with cerebral venous sinus thrombosis (CVST) exhibits significant heterogeneity, and the mechanisms underlying these distinct trajectories remain poorly understood.

**Objective:**

This study aimed to identify distinct long-term trajectories of functional outcome in CVST patients and to investigate their baseline predictors.

**Methods:**

In a longitudinal cohort of 127 CVST patients with complete follow-up data, we employed latent class mixed models (LCMM) to analyze repeated measures of the modified Rankin Scale (mRS) over time. The optimal number of trajectory classes was determined using established information criteria. Multivariable logistic regression was then used to identify baseline factors independently associated with class membership.

**Results:**

A two-trajectory model best characterized the heterogeneity in long-term outcomes. Class 1 (the “Poor-Recovery” Class, *n* = 66, 52%) was characterized by older age, poorer venous collateral circulation, higher D-dimer levels, more severe initial brain parenchymal changes, and a slower, incomplete functional recovery. Class 2 (the “Favorable-Recovery” Class, *n* = 61, 48%) consisted of younger patients with better collateral circulation and a rapid, near-complete recovery. Multivariable analysis confirmed that increasing age [odds ratio (OR): 1.11 per year, 95% confidence interval (CI): 1.06–1.18, *p* = 0.0001] was an independent risk factor, while a higher venous collateral circulation score (indicating better collateral function; OR: 0.255 per point, 95% CI: 0.108–0.540, *p* = 0.0008) was a strong protective factor against belonging to the “Poor-Recovery” class.

**Conclusion:**

CVST patients follow one of two distinct long-term functional trajectories, which are predominantly driven by age and the status of the venous collateral circulation. These findings provide a mechanism-based framework for prognosis and highlight the potential for early risk stratification, paving the way for more personalized patient management.

## Introduction

Cerebral venous sinus thrombosis (CVST) is a distinct cerebrovascular disorder characterized by thrombotic occlusion of the cerebral veins and/or dural sinuses, accounting for approximately 0.5–1% of all strokes (Alghamdi et al., 2022; Nguyen et al., 2024). Unlike arterial strokes, CVST predominantly affects younger adults and presents with a highly variable clinical spectrum, ranging from isolated headache to severe neurological deficits, coma, and death (Javed et al., 2018; Alet et al., 2020). Over the past decades, advancements in diagnostic imaging and the widespread application of anticoagulant therapy have significantly reduced the acute-phase mortality of CVST. Consequently, research and clinical focus have progressively shifted towards understanding the long-term functional outcomes and quality of life of survivors.

A critical challenge in the long-term management of CVST is the profound heterogeneity in neurological recovery. While a majority of patients achieve functional independence [modified Rankin Scale (mRS) score of 0–2], a substantial proportion experiences persistent cognitive impairment, fatigue, or residual disability, profoundly impacting their personal and professional lives (Saroja et al., 2020; Kajbaf et al., 2021). The underlying mechanisms driving these divergent recovery trajectories remain poorly elucidated. Current prognostic models primarily rely on baseline clinical and radiological factors, such as coma, intracranial hemorrhage, or malignancy, to predict a dichotomized outcome at a single time point (Ferro and de Aguiar Sousa, 2019; Pizzi et al., 2016). However, this static, group-averaged approach fails to capture the dynamic and multidimensional nature of post-CVST neurological evolution. It obscures the possibility that distinct subpopulations of patients may follow characteristically different functional trajectories over time, each governed by a unique set of biological and pathological mechanisms.

To unravel this complexity, a methodological shift from variable-centered to person-centered analytical approaches is required. Latent class mixed models (LCMM) represent a powerful statistical framework that addresses this need (Hoekstra and Twisk, 2015; Leiby et al., 2014; Zhang and Ip, 2014). Unlike conventional mixed models that estimate an average trajectory for the entire population, LCMM can identify unobserved (latent) subpopulations of individuals who share similar patterns of change in a longitudinal outcome (Pei et al., 2024; Badiya et al., 2020), such as the mRS. This approach has proven invaluable in characterizing recovery heterogeneity in other neurological conditions, such as traumatic brain injury and ischemic stroke (Wickwire et al., 2022; Gardner et al., 2019), but its application to CVST remains largely unexplored.

Elucidating these distinct trajectories is not an endpoint in itself; it is a crucial first step toward a “precision prognosis” paradigm. The subsequent, and equally important, step is to uncover the mechanisms—the specific clinical and neuroimaging factors—that predispose patients to a particular recovery path. Key postulated mechanisms include the brain’s capacity for compensation, reflected in collateral circulation status, the extent of initial parenchymal injury (e.g., hemorrhage, infarction), and the systemic thrombotic burden, measured by biomarkers like D-dimer (Domitrz et al., 2020; Sun et al., 2023). An integrative model that links trajectory subgroups with their underlying mechanisms is urgently needed to move beyond descriptive prognosis and towards predictive, actionable insights.

Therefore, this longitudinal study aims to: (1) identify and characterize distinct long-term (≥1 year) neurological functional trajectories in a cohort of CVST patients using LCMM; and (2) construct an integrated dynamic prognostic model by investigating the associations between these trajectory subgroups and a comprehensive set of baseline clinical, radiological, and laboratory factors. We hypothesize that at least two discrete mRS trajectory classes exist—one favoring favorable recovery and another associated with poor outcome—and that these classes are differentially predicted by a combination of age, collateral circulation status, and patterns of brain parenchymal injury.

## Methods

### Study design and population

This retrospective longitudinal study consecutively enrolled 127 patients with cerebral venous sinus thrombosis (CVST) who were diagnosed and treated between January 2017 and June 2024. The diagnosis of CVST was confirmed by magnetic resonance imaging (MRI) combined with magnetic resonance venography (MRV), in accordance with the Chinese guidelines for the diagnosis and management of intracranial venous thrombosis (Fan et al., 2020).

Inclusion criteria comprised: (1) complete clinical and imaging data; (2) receipt of standardized, full-course anticoagulant therapy; and (3) availability of follow-up data without loss to follow-up or refusal to participate. Patients were excluded if they presented with other severe intracranial pathologies unrelated to CVST, had pulmonary embolism, deep vein thrombosis, or thrombosis in other sites, had received anticoagulant therapy prior to this study, suffered from severe comorbidities in other organs, or had undergone interventional procedures such as sinus thrombectomy, balloon angioplasty, or decompressive craniectomy.

The study was approved by the Institutional Review Board and written informed consent was obtained from all patients or their legal guardians.

### Data collection

Demographic characteristics, clinical presentation, disease course (classified as acute, subacute, or chronic), predisposing factors, and laboratory parameters (including D-dimer concentration measured on admission) were meticulously retrieved from electronic medical records. All patients underwent a standardized follow-up protocol, with functional assessments using the modified Rankin Scale (mRS) recorded at five time points: admission, discharge, 3 months, 6 months, and at the last outpatient visit (up to 62 months).

### Neuroimaging analysis

Initial and follow-up MRI/MRV images were independently reviewed by two experienced radiologists. Discrepancies were resolved through consensus with a senior radiologist holding associate chief physician qualifications or higher. The following imaging parameters were assessed: (1) Brain parenchymal changes: Categorized as none, hemorrhage, infarction, or mixed patterns. (2) Venous collateral circulation: Graded using the Sheth collateral circulation scale (Sedlacik et al., 2010; Sheth and Liebeskind, 2014; Sheth et al., 2018): 0 (no venous drainage in the affected territory), 1 (drainage present but not connected to the occluded sinus), or 2 (drainage present and connected to the occluded sinus). A higher score indicates superior compensatory capacity. (3) Venous recanalization: Assessed and classified as none, partial, or complete recanalization.

### Treatment protocol

Upon diagnosis, patients-initiated anticoagulation therapy with an intravenous heparin pump or subcutaneous low-molecular-weight heparin for 7–10 days. The dosage of low-molecular-weight heparin was weight-adjusted (90–100 AxaIU/kg, twice daily). Oral anticoagulants were introduced once the target international normalized ratio was achieved. Concomitant management, including intracranial pressure reduction, fluid resuscitation, antiepileptic, and anti-infective therapies, was administered as clinically indicated.

### Statistical analysis

Continuous variables are presented as mean ± standard deviation, and categorical variables as frequencies (percentages). Group comparisons for baseline characteristics were performed using Student’s *t*-test or chi-squared test, as appropriate.

The core analysis employed latent class mixed models (LCMM) using the lcmm package (version 1.9.5) in R software (R foundation, version 4.1.0) to identify distinct longitudinal trajectories of the mRS score over time. The mRS score was modeled as an ordinal outcome using a cumulative probit link function (link = “thresholds”). Models specifying 1, 2, and 3 latent classes were fitted and compared. Each model included a fixed effect of time and a random intercept per subject (ID). The optimal number of latent classes was determined by comparing models using the Bayesian information criterion (BIC) and the Akaike information criterion (AIC), with lower values indicating a better balance between model fit and parsimony.

Following the identification of the optimal trajectory classes, patients were assigned to their most probable class based on the model’s posterior probabilities. To identify baseline factors independently associated with class membership, a multivariable logistic regression model was constructed, with class membership as the dependent variable and key demographic, clinical, and imaging characteristics as covariates. Results are reported as odds ratios (ORs) with 95% confidence intervals (CIs). A two-sided *p*-value <0.05 was considered statistically significant. All analyses were conducted using R software (version 4.1.0).

## Results

### Patient characteristics and latent class model selection

A total of 127 CVST patients with complete longitudinal mRS assessments (with 5 time points per patient) were included in the final analysis. The baseline demographic, clinical, and radiological characteristics of the overall cohort are summarized in Table 1. The mean age was 45.99 ± 16.8 years, and 54% of the patients were male.

**Table 1:** Baseline demographic, clinical, and radiological characteristics of the overall cohort (*N* = 127).

Variables	Overall (*N* = 127)
Age	45.99 ± 16.8
Gender	
Male	68 (54%)
Female	59 (46%)
Disease course	
Acute	45 (35%)
Subacute	65 (51%)
Chronic	17 (13%)
Focal neurological deficits	
No	63 (50%)
Yes	64 (50%)
Brain parenchymal changes	
No	33 (26%)
Hemorrhage	26 (20%)
Infarction	40 (31%)
Mixed	28 (22%)
Collateral circulation score	
0	31 (24%)
1	44 (35%)
2	52 (41%)
D-dimer concentration (μg/mL)	3.31 ± 2.4

Data are presented as mean ± standard deviation for continuous variables and *n* (%) for categorical variables. CVST, cerebral venous sinus thrombosis.

The model fit indices for the latent class mixed models (LCMM) are presented in Table 2. The 2-class model demonstrated a significantly better fit compared to the 1-class model, as evidenced by the lower Akaike information criterion (AIC: 1911.278 vs. 2128.161) and Bayesian information criterion (BIC: 1942.564 vs. 2150.915). The 3-class model failed to converge properly, yielding an implausible log-likelihood and exceedingly high AIC/BIC values, indicating overfitting or non-identification. Therefore, the 2-class solution was selected as the optimal model for characterizing the heterogeneity in mRS trajectories. The posterior classification quality for the 2-class model was excellent, with a high mean probability of assignment to the designated class (0.975 for class 1 and 0.947 for class 2).

**Table 2:** LCMM model comparison for CVST mRS trajectories.

Variables	Classes	LogLikelihood	AIC	BIC
1-class	1	−1056.081	2128.161	2150.915
2-class	2	−944.639	1911.278	1942.564
3-class	3	−1.00 × 10^9^	2,000,000,028	2,000,000,068

The 2-class model was selected as optimal based on the lowest Akaike (AIC) and Bayesian (BIC) information criteria. The 3-class model failed to converge, resulting in implausible fit indices, indicating overfitting. LCMM, latent class mixed model.

### Distinct functional trajectories and baseline profiles of the latent classes

**Figure 1:**
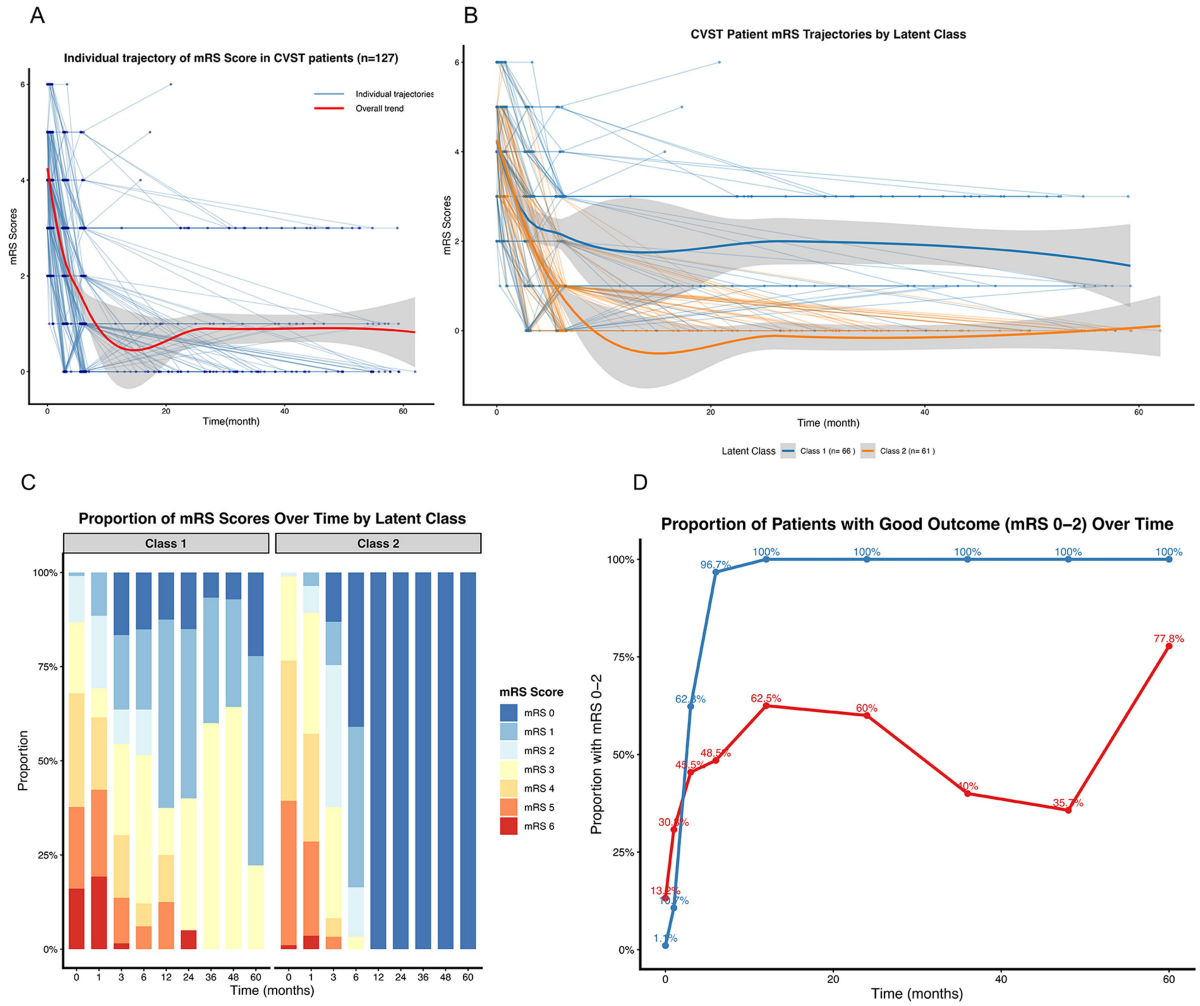
Heterogeneity in long-term functional outcomes and identification of distinct recovery trajectories in CVST. **(A)** Spaghetti plot of individual modified Rankin Scale (mRS) trajectories for all 127 patients, demonstrating the overall heterogeneity in recovery courses. **(B)** Model-estimated mean trajectories for the two latent classes identified by latent class mixed modeling. Class 1 represents the “Poor-Recovery” trajectory (*n* = 66), characterized by higher initial disability and slower improvement. Class 2 represents the “Favorable-Recovery” trajectory (*n* = 61), characterized by rapid and sustained recovery. Shaded areas represent 95% confidence intervals. **(C)** Stacked bar charts showing the distribution of mRS scores (0–5) over time within each latent class, illustrating the dynamic evolution of functional status. **(D)** The proportion of patients with a good functional outcome (defined as mRS score 0–2) over time. The trajectories diverge early, with class 2 achieving near-universal good outcomes, in contrast to class 1.

The individual trajectories of all 127 CVST patients illustrated considerable heterogeneity in functional recovery, with no single pattern characterizing the entire cohort (Figure 1A). The LCMM analysis resolved this heterogeneity into two discrete trajectory classes. Class 1 (*n* = 66, 52%) was characterized by a “Poor-Recovery” trajectory, featuring higher initial mRS scores and a slower, less complete functional improvement. In contrast, class 2 (*n* = 61, 48%) demonstrated a “Favorable-Recovery” trajectory, with lower initial disability and a rapid, sustained recovery (Figure 1B). The evolution of mRS score distributions over time further highlighted the stark contrast between the classes, with class 2 shifting rapidly towards lower scores (0–1) while class 1 retained a higher proportion of elevated scores (3–5) throughout follow-up (Figure 1C). Consequently, the proportion of patients achieving a good functional outcome (mRS 0–2) diverged sharply and early between the classes, with nearly all patients in class 2 reaching this benchmark and sustaining it, compared to a significantly lower and slower-improving proportion in class 1 (Figure 1D).

The comparison of baseline characteristics between the two latent classes is detailed in Table 3. Patients in the Poor-Recovery class (class 1) were significantly older than those in the Favorable-Recovery class (class 2) (57.17 ± 12.2 years vs. 33.9 ± 12.1 years, *p* < 0.001). Furthermore, class 1 had a significantly higher prevalence of focal neurological deficits (67% vs. 33%, *p* < 0.001) and more severe brain parenchymal changes on initial neuroimaging, with a notably higher proportion of mixed patterns (33% vs. 9%, *p* < 0.001). Patients in the Poor-Recovery class had significantly worse venous collateral circulation scores (*p* < 0.001), with a predominance of lower scores (0 or 1) indicating inferior collateral function. Accordingly, the systemic thrombotic burden, reflected by the D-dimer concentration on admission, was significantly higher in class 1 (4.46 ± 2.4 μg/mL vs. 2.07 ± 1.7 μg/mL, *p* < 0.001). No significant differences were observed between the two classes in terms of gender distribution or disease course.

**Table 3:** Comparison of baseline characteristics between the two identified latent classes.

Variables	Group		*t*/*χ*^2^	*p*-value
	Class 1 (*N* = 66)	Class 2 (*N* = 61)		
Age	57.17 ± 12.2	33.9 ± 12.1	10.794	**<0.001**
Gender			0.429	0.513
Male	33 (50%)	35 (57%)		
Female	33 (50%)	26 (43%)		
Disease course			0.968	0.616
Acute	26 (39%)	19 (31%)		
Subacute	32 (48%)	33 (54%)		
Chronic	8 (12%)	9 (15%)		
Focal neurological deficits			13.232	**<0.001**
No	22 (33%)	41 (67%)		
Yes	44 (67%)	20 (33%)		
Brain parenchymal changes			20.972	**<0.001**
No	7 (11%)	26 (43%)		
Hemorrhage	14 (21%)	12 (20%)		
Infarction	23 (35%)	17 (28%)		
Mixed	22 (33%)	6 (9%)		
Collateral circulation score			37.773	**<0.001**
0	43 (65%)	9 (14%)		
1	18 (27%)	26 (43%)		
2	5 (8%)	26 (43%)		
D-dimer concentration (μg/mL)	4.46 ± 2.4	2.07 ± 1.7	6.406	**<0.001**

The “Poor-Recovery” class (class 1, *n* = 66) and “Favorable-Recovery” class (class 2, *n* = 61) are defined by their distinct longitudinal mRS trajectories. Data are presented as mean ± standard deviation or *n* (%). Group comparisons were performed using Student’s *t*-test (reported as *t*-statistic) for continuous variables and chi-squared test (reported as *χ*^2^ statistic) for categorical variables. *p*-values in bold indicate statistical significance (*p* < 0.05).

### Independent predictors of latent class membership

**Figure 2:**
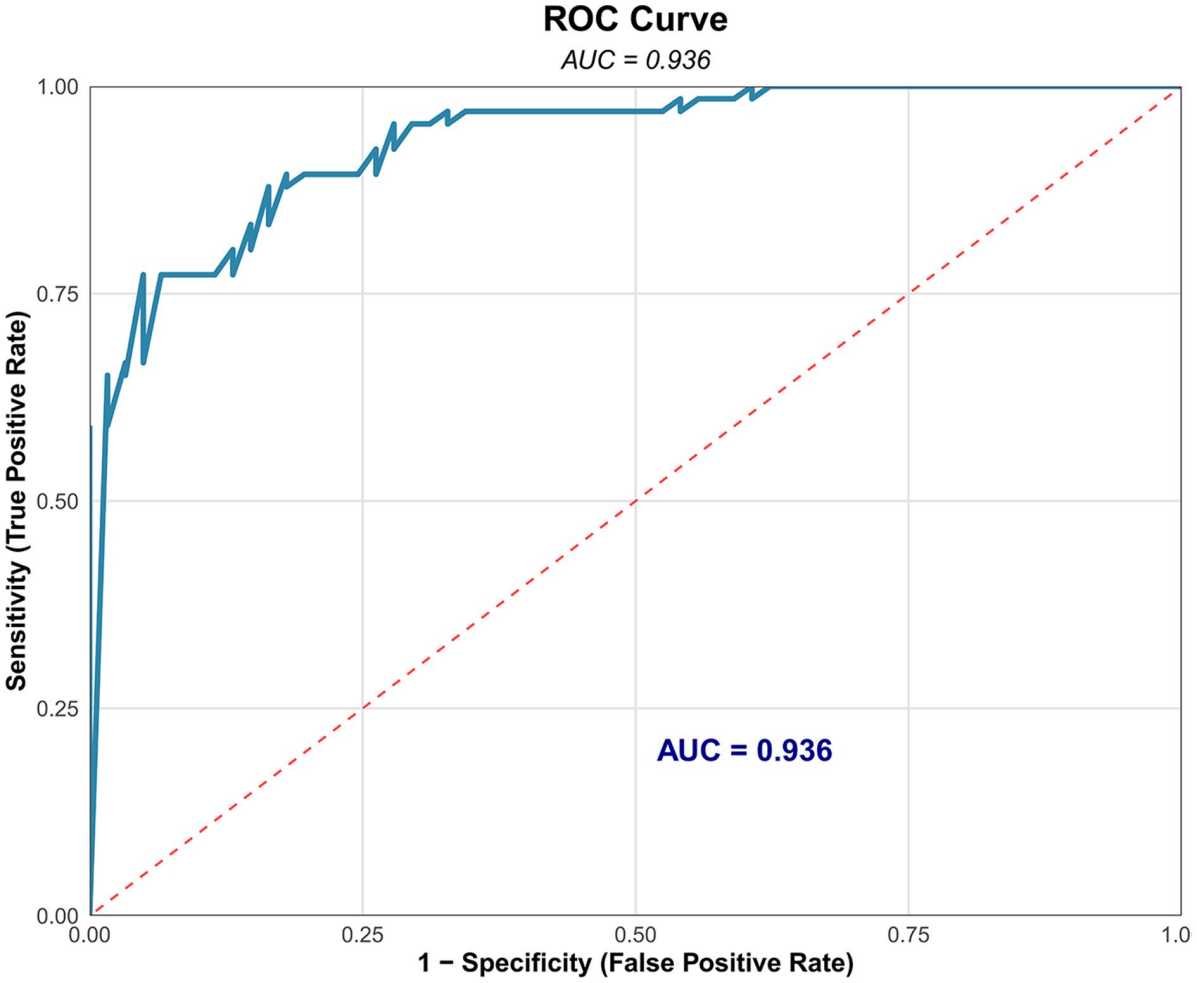
Receiver operating characteristic (ROC) curve for the multivariable prediction model. The ROC curve evaluates the performance of the logistic regression model (incorporating age and collateral circulation score) in discriminating the “Poor-Recovery” class. The area under the curve (AUC) of 0.936 indicates outstanding model discriminative ability.

The results of the multivariable logistic regression analysis, identifying factors independently associated with membership in the Poor-Recovery class (class 1), are shown in Table 4. After adjusting for other covariates, increasing age [odds ratio (OR): 1.11 per year, 95% confidence interval (CI): 1.06–1.18, *p* = 0.0001] was an independent risk factor for poor recovery, while a higher venous collateral circulation score (indicating better collateral function; OR: 0.255 per point, 95% CI: 0.108–0.540, *p* = 0.0008) was a strong protective factor, with an AUC of 0.936 (Figure 2). In this model, D-dimer concentration, the presence of focal neurological deficits, and the pattern of brain parenchymal changes were not independently associated with class membership.

**Table 4:** Multivariable logistic regression analysis of factors associated with “Poor-Recovery” class membership.

Variables	Estimate	*Z* value	OR	95% CI lower	95% CI upper	*p*-value
Age	0.1064	4.0166	1.1122	1.0601	1.1777	**0.0001**
D-dimer concentration (μg/mL)	0.0964	0.6870	1.1011	0.8335	1.4598	0.4921
Focal neurological deficits	0.1892	0.3042	1.2083	0.3444	4.0611	0.7610
Brain parenchymal changes	0.3961	1.4835	1.4860	0.8866	2.5576	0.1379
Collateral circulation score	−1.3665	−3.3701	0.255	0.108	0.540	**0.0008**

The model demonstrates excellent discriminative ability (AUC = 0.936). Results are presented as odds ratios (OR) with 95% confidence intervals (CI). CI, confidence interval; OR, odds ratio. *p*-values in bold indicate statistical significance (*p* < 0.05).

## Discussion

This longitudinal study provides novel evidence for the existence of distinct long-term functional trajectories in patients with cerebral venous sinus thrombosis, thereby advancing our understanding of the heterogeneous nature of neurological recovery in this condition. By applying latent class mixed modeling (LCMM) to repeated mRS assessments over an extended period, we identified two discrete recovery patterns: a “Favorable-Recovery” trajectory (class 2) and a “Poor-Recovery” trajectory (class 1). Crucially, we further demonstrated that these trajectories are not random but are robustly predicted by a combination of age and the integrity of the venous collateral circulation, offering key insights into the underlying mechanisms.

Our most salient finding is the identification of two latent classes with divergent prognostic paths. The “Poor-Recovery” class, comprising over half of our cohort, was characterized by an older age at onset, a higher burden of initial neurological deficits and parenchymal injury, and a sluggish recovery course. In contrast, the “Favorable-Recovery” class was typified by younger patients with less severe initial imaging findings and a rapid, near-complete functional restoration. This delineation moves beyond traditional, static outcome measures and captures the dynamic process of recovery, aligning with the growing recognition of outcome heterogeneity in cerebrovascular diseases (Phan et al., 2017). The excellent classification quality of our model underscores that these are not arbitrary groupings but represent biologically and clinically distinct patient phenotypes.

The mechanistic insights from our study are twofold, centering on the two independent predictors identified in the multivariable model. First, increasing age emerged as a powerful determinant of an unfavorable trajectory. This finding is consistent with the broader stroke literature, where advanced age is consistently associated with worse outcomes, potentially due to reduced neuronal plasticity, increased comorbidities, and lower brain reserve capacity (Costigan et al., 2009; Wang et al., 2022; Hu et al., 2024; Anatürk et al., 2021). In the specific context of CVST, an older brain may possess a diminished ability to compensate for the acute insult of venous hypertension and ischemia, leading to more extensive injury and a hampered recovery process.

Second, and perhaps more specific to CVST pathophysiology, we found that a lower venous collateral circulation score was independently associated with the “Poor-Recovery” trajectory. The cerebral venous system possesses a rich collateral network, and its efficacy is critical in mitigating the hemodynamic consequences of sinus occlusion. A robust collateral circulation (score of 2) likely facilitates the redistribution of venous outflow, thereby limiting venous congestion, intracranial pressure elevation, and subsequent parenchymal damage (hemorrhage/infarction). Our results provide empirical support for this, showing that a robust collateral circulation (score of 2) was far more prevalent in the “Favorable-Recovery” class (43%) compared to the “Poor-Recovery” class (8%, *p* < 0.001). The strong independent effect of collateral status after adjusting for age and parenchymal injury suggests that it is a primary mechanism influencing the recovery trajectory, rather than merely a secondary phenomenon.

It is noteworthy that while univariate analysis showed significant associations for focal deficits, brain parenchymal changes, and D-dimer levels with class membership, these factors were not independent in the multivariable model. This indicates that their influence is likely mediated through or confounded by the core mechanisms of age and collateral capacity. For instance, the extent of parenchymal injury may be a downstream consequence of poor collaterals, and its prognostic information is thus captured by the collateral score itself.

Our findings must be contextualized within the landscape of existing prognostic tools for CVST. Clinical prediction scores, such as those by Ferro et al. (2004), Bushnaq et al. (2018), and more recently Li et al. (2023), have been instrumental in stratifying patients based on baseline clinical and radiological factors. Notably, age has emerged as a consistent and strong predictor across these models, a finding robustly corroborated by our LCMM analysis. However, a critical examination reveals that these established scores predominantly incorporate factors such as coma, intracranial hemorrhage, malignancy, or infection, which often represent the consequences of the thrombotic event or severe systemic states. In contrast, our model highlights venous collateral circulation—a factor reflecting the brain’s intrinsic compensatory capacity at the onset of venous occlusion. While thrombus burden scores, exemplified by the work of Wang et al. (2023), provide a valuable quantification of the extent of sinus involvement, they primarily describe the anatomical extent of the insult. Our results suggest that the functional status of venous collaterals may act as a key moderator between thrombus burden and parenchymal injury, thereby influencing the ultimate recovery trajectory. The fact that collateral status remained an independent predictor after adjusting for age and parenchymal changes underscores its role as a primary, rather than secondary, pathophysiological determinant. Therefore, our study extends the existing paradigm by proposing that integrating a measure of physiological reserve (collateral circulation) with a core demographic factor (age) may offer a more mechanism-oriented framework for “precision prognosis,” complementing the established clinical-anatomical models.

Several scales exist to grade venous collateral circulation. We selected the Sheth score for this analysis based on three principal considerations (Sheth and Liebeskind, 2014; Sheth et al., 2018). First, its simplicity and reproducibility—a straightforward three-tier ordinal scale (0–2)—make it highly feasible for consistent application in routine clinical radiology practice, a key factor for potential future translation. Second, it has established validity and prognostic correlation specifically within the CVST population, directly linking collateral status to clinical outcomes (Sheth et al., 2018). Third, compared to more complex quantitative measures, this scale efficiently captures the functional capacity of the venous collateral network (absent, present but non-connected, present and connected), which aligns with our aim to assess a patient’s intrinsic physiological compensatory reserve at admission.

### Clinical implications

Our findings have direct clinical relevance. The integration of age and a simple, radiological assessment of venous collaterals could form the basis of an early risk-stratification tool. Identifying patients at high risk for a “Poor-Recovery” trajectory at admission could enable closer monitoring, more aggressive management of intracranial pressure, and potentially guide the intensity and duration of rehabilitation efforts. Furthermore, the collateral circulation may represent a potential therapeutic target, where future interventions aimed at promoting or supporting venous collateral flow could improve outcomes.

### Limitations

Several limitations of our study merit consideration. First, the single-center, retrospective design may limit the generalizability of our findings, and prospective validation in a multi-center cohort is warranted. Second, while our sample size was sufficient for the LCMM analysis, it may have limited the power to detect weaker independent predictors in the regression model. Third, the LCMM approach, while powerful, relies on certain statistical assumptions, and the trajectories are influenced by the variables included in the model. We did not explore all potential covariates, such as detailed thrombophilia profiles or specific rehabilitation protocols, which could provide further insights. Finally, the assessment of collaterals, though performed by experienced radiologists, would benefit from future standardization and quantitative validation.

## Conclusion

In conclusion, this study demonstrates that CVST patients follow one of two distinct long-term functional trajectories, which are predominantly determined by the interplay between patient-specific reserve (age) and disease-specific compensatory capacity (venous collateral circulation). This “precision prognosis” framework enhances our ability to predict recovery patterns and provides a mechanistic basis for future research aimed at improving outcomes in this patient population.

## Data Availability

The raw data supporting the conclusions of this article will be made available by the authors, without undue reservation.
